# Enhanced Astrocytic Nitric Oxide Production and Neuronal Modifications in the Neocortex of a NOS2 Mutant Mouse

**DOI:** 10.1371/journal.pone.0000843

**Published:** 2007-09-05

**Authors:** Yossi Buskila, Yasmin Abu-Ghanem, Yifat Levi, Arie Moran, Ettie Grauer, Yael Amitai

**Affiliations:** 1 Department of Physiology, Faculty of Health Sciences, Ben-Gurion University, Beer-Sheva, Israel; 2 Department of Pharmacology, Israel Institute for Biological Research, Ness-Ziona, Israel; National Institutes of Health, United States of America

## Abstract

**Background:**

It has been well accepted that glial cells in the central nervous system (CNS) produce nitric oxide (NO) through the induction of a nitric oxide synthase isoform (NOS2) only in response to various insults. Recently we described rapid astroglial, NOS2-dependent, NO production in the neocortex of healthy mice on a time scale relevant to neuronal activity. To explore a possible role for astroglial NOS2 in normal brain function we investigated a NOS2 knockout mouse (B6;129P2-*Nos2^tm1Lau^*/J, Jackson Laboratory). Previous studies of this mouse strain revealed mainly altered immune responses, but no compensatory pathways and no CNS abnormalities have been reported.

**Methodology/Principal Findings:**

To our surprise, using NO imaging in brain slices in combination with biochemical methods we uncovered robust NO production by neocortical astrocytes of the NOS2 mutant. These findings indicate the existence of an alternative pathway that increases basal NOS activity. In addition, the astroglial mutation instigated modifications of neuronal attributes, shown by changes in the membrane properties of pyramidal neurons, and revealed in distinct behavioral abnormalities characterized by an increase in stress-related parameters.

**Conclusions/Significance:**

The results strongly indicate the involvement of astrocytic-derived NO in modifying the activity of neuronal networks. In addition, the findings corroborate data linking NO signaling with stress-related behavior, and highlight the potential use of this genetic model for studies of stress-susceptibility. Lastly, our results beg re-examination of previous studies that used this mouse strain to examine the pathophysiology of brain insults, assuming lack of astrocytic nitrosative reaction.

## Introduction

Nitric oxide (NO) is produced in the brain by neurons, astroglia and endothelial cells, and is known to participate in diverse signaling pathways. Three different isoforms of the enzyme nitric oxide synthase (NOS) synthesize NO from L-arginine, and their activity depends on the setting and the cell type involved. For example, neurons expressing the neuronal NOS isoform (NOS1) are capable of rapid release of small amounts of NO serving as neurotransmitter [1], [2]. On the other hand, astroglial NO production has been demonstrated mainly as a reaction to various detrimental stimuli such as ischemia or inflammation, through the activity of a stress-induced NOS isoform (NOS2) that can produce large amounts of NO, but on a much slower time scale [e.g. 3]–[7]. Using NO imaging in brain slices we recently demonstrated NOS2-dependent astroglial NO production, which occurred on a fast time scale (seconds) and did not involve de-novo protein synthesis [8]. These data raised, for the first time, the possibility of the involvement of astroglial-derived NO in physiological brain activity.

To test for a possible role for astroglial, NOS2-dependent NO in normal brain function, we evaluated a NOS2 knockout mouse (B6;129P2-*Nos2^tm1Lau^*/J, Jackson Laboratory, http://jaxmice.jax.org/strain/002596rf.html). In this mouse strain, the fragment containing the calmodulin-binding domain of NOS2 was replaced by the neomycin resistance gene [9]. Homozygous NOS2 mutant mice are fertile, and display no developmental defects or abnormalities of blood composition. Over the past ten years, more than 100 published studies have used these NOS2 mutants to explore the role of the enzyme in various organ systems. It has been reported that these mice have no serum NO response under conditions of immune challenge, their macrophages do not produce NO in culture, and they exhibit altered responses to various systemic infections, though they are not considered immune-compromised [9]–[11]. No compensatory pathway has been identified in this mouse strain untill now.

Only a few studies have explored some aspects of brain function in these mutants. Almost all these studies used the mutant in various models for brain pathologies such as ischemia, Alzheimer's or Parkinson's disease, focusing on the role of NOS2 induction in the long-term outcome of the insult. The mutants were compared to control mice, assuming they lack astroglial NO production [e.g. 12]–[16]. The behavior of these mutants received little attention, probably due to the current conviction that maintains that NOS2 does not participate in normal brain activity.

In the current study we demonstrate robust NO production by astroglia in the mutants' neocortex, suggestive of a constitutive alternative pathway. The data also imply that the mutation lead to neuronal modifications, and a distinct behavioral phenotype. Some of these data have been published in an abstract format (Buskila et al., SfN abst., Atlanta 2006).

## Results

### Mutants' astrocytes exhibit NOS activity

**Figure 1:**
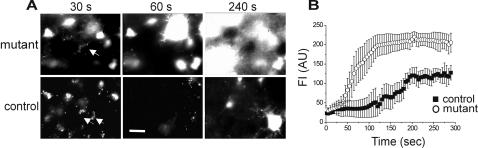
DAF-2DA fluorescence in mutant mice is faster and stronger. (A) Slices from mutant and control mice were pre-incubated in DAF-2DA (2 µM) for 10 minutes, and imaged with a fluorescent light source. Example images are displayed at three time points following the start of illumination (marked above). Arrowheads at 30 s point at punctate staining of putative neurons. Note the larger and steadily increasing number of diffusely-fluorescing, putative astroglia in slices from the mutant mouse. Scale bar = 25 µm. (B) Summary diagram of diffuse DAF-2DA fluorescent changes over time. Data are displayed as mean FI±S.E.M from identified single cells (control-26 cells from 4 slices, open circles; mutants–58 cells from 4 slices, closed squares).

We first examined whether the NOS2 mutation eliminated astroglial NO production as expected. We incubated cortical slices from mutant and control mice in the NO indicator diaminofluorescein–2 diacetate (DAF-2DA) to image NO-producing cells. The fluorescence intensity (FI) of this indicator is expected to roughly correlate with the concentration of NO and peroxinitrite and hence, with NOS activity [17], [18]. The staining pattern observed under the fluorescent light in slices from control mice was identical to previously described DAF-2DA fluorescence in slices from CD1 mice [8]. Briefly, punctate, putative neuronal staining appeared almost immediately and decayed within 60 to 90 seconds. Putative astroglia staining appeared later, at variable times from the start of illumination (usually>120 sec), displaying bright diffuse staining (Fig 1A). In contrast, slices from mutant mice exhibited widespread cellular fluorescence immediately after opening the fluorescence shutter. The fluorescence pattern was mixed, and both punctate and diffuse staining could be observed. Longer illumination periods led to a rapid increase in the number of diffusely fluorescent cells resulting in considerable overlap and making it impossible to differentiate single cell somata (Fig 1A). The non-selective NOS inhibitor L-NAME (1mM) abolished all fluorescent responses in slices from both mutant (n = 4 slices, 2 animals) and control (n = 4 slices, 2 animals) mice, corroborating it was due to NOS activity.

Fig 1B displays FI analysis of identified cells over time, demonstrating faster and more intense fluorescent response in slices from mutant compared with control mice. Since FI of single diffusely-stained cells reached saturation levels in both control and mutant mice, it can be deduced that in mutant slices, the rapid increase in FI was caused mainly by a higher number of cells participating rapidly and simultaneously in NO production.

**Figure 2:**
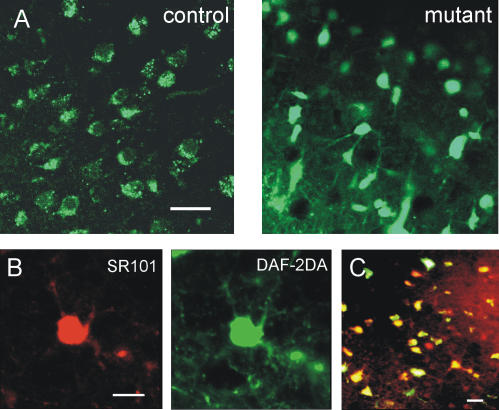
Diffusely-stained astrocytes are prevalent in confocal images from mutant mice. (A) Example of confocal images from DAF-2DA incubated neocortical slices. Punctate, putative neuronal staining is dominating in slices from control mice (left), and diffuse fluorescence is dominating in the mutant's slice (right). Scale bar = 25 µm. (B) Diffuse DAF-2DA staining (green) is co-localized with the specific astrocytic marker SR101 (red). Examples from confocal images of a single cell are displayed. Scale bar = 10 µm. (C) Merging image of SR101 and DAF-2DA staining (projection of four 5 µm thick images). Cells which stain with both dyes appear in yellow. Over 95% of the cells displayed co-localization (4 slices, 2 animals). Scale bar = 20 µm.

Previous data suggested that the reason for the delayed astroglial DAF-2DA fluorescence in CD1 mice is an unidentified activation step which was triggered by phototoxic neuronal death [8]. In agreement with this idea, diffuse astroglial staining was rarely observed in DAF-2DA loaded slices from control mice when imaged with a confocal microscope. In contrast, cells exhibiting diffuse fluorescence pattern were readily noticeable and widespread under the confocal microscope in slices from mutant mice (n = 8 slices, 3 animals, Fig 2A). Thus, the diffuse fluorescent response in the mutant did not require any preceding event, but rather the NOS isoform in these cells was probably constitutively active. In addition, diffuse DAF-2DA fluorescence in mutant slices was almost fully co-localized with the specific astrocytic marker SR101 [19] (Fig 2B,C), establishing that the pattern of DAF-2DA staining has not been altered in the mutant, and the cells exhibiting diffuse fluorescence are astrocytes.

**Figure 3:**
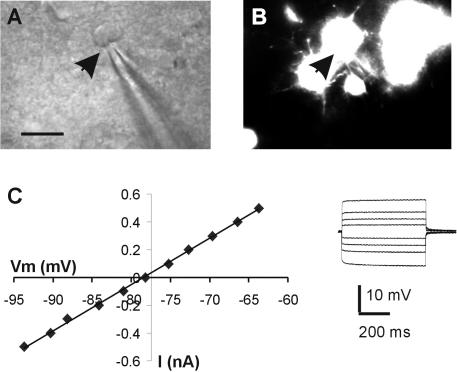
Electrophysiological properties of diffusely fluorescent cells in mutant slices are characteristic of astroglia. (A) IR/DIC image from a mutant slice displays the recording pipette and the cell's soma. The arrowhead points to the location of the pipette tip. Scale bar = 20 µm. (B) A fluorescent image of the same region as in (A) reveals several diffusely fluorescent cells. The arrowhead position is the same as in (A). (C) A series of current pulses at 0.1 nA increments were delivered through the recording pipette (inset). A plot of the current pulse intensity (I) vs. the voltage deflection (Vm) reveals linear relationship characteristic of astroglia.

Whole-cell recordings from diffusely stained cells in slices from control mice disclosed low membrane resistance (Ri 30.6±23.2 MΩ, n = 6) and passive membrane properties characteristic of astroglia. We also recorded from diffusely-stained cells in mutant slices and examined their electrophysiological properties: all the recorded cells (n = 6) revealed low input resistance (average 34.1±19.4 MΩ), a linear I-V relationship and lack of action potential firing upon depolarization (Fig 3). There were no significant differences between astrocytes from the mutant and control mice in their electrophysiological properties, and these were similar to previously published data on astrocytes in CD1 mice [8]. Taken together, these findings indicate that astrocytes in the mutant mice express an active NOS isoform.

### NOS activity in mutant astroglia is ca^2+^-dependent

**Figure 4:**
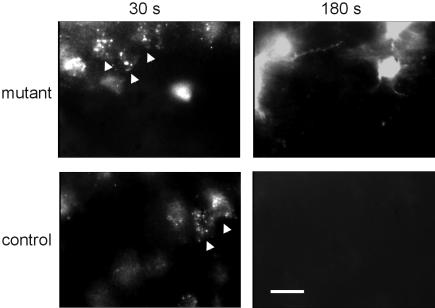
Mutant astroglial NOS activity is unaffected by NOS2 inhibitor. Example images from slices incubated in the selective NOS2 inhibitor 1400W (3 µM) for at least 30 minutes. At 30 seconds from the beginning of illumination (left panels), neuronal punctate fluorescence (arrow heads) was abundant in slices from either mutant or control mice. At 180 seconds (right panels), astrocytic diffuse fluorescence is abolished in slices from control mice, but not in mutant slices. Images were taken with ND4 filter to slow the response and allow for cells' separation. Scale bar = 20 µm.

To identify the isoform responsible for the augmented fluorescent response in mutant mice we used specific NOS inhibitors. An inhibitor of constitutive NOS isoforms, L-NNA (1 µM), abolished all fluorescent reaction in slices from both mutant (n = 5 slices, 2 animals) and control mice (n = 5 slices, 2 animals). 1400W is considered today the most selective NOS2 inhibitor, as it inhibits NOS2 with 5000- and 200-folds greater potency than NOS3 and NOS1, respectively [20]. Incubating brain slices for 30 minutes in a ringer solution containing 1400W (3 µM) had no effect on the punctate, neuronal fluorescence in slices from either mutant or control mice (Fig. 4, 30 sec). On the other hand, 1400W abolished the astrocytic diffuse fluorescence in all slices from control (n = 6 slices, 2 animals), but not in slices from mutant mice, where multiple diffusely-stained astrocytes were clearly visible (Fig 4, 180sec, n = 7 slices, 3 animals). These findings are consistent with the premise that the NOS2 protein in astroglia of mutant mice has been modified, and its pharmacological sensitivity to the drug has been altered.

NOS2 activity is considered independent of intracellular Ca^2+^ concentration. Knowing that the mutation was directed at the calmodulin-binding domain of the protein, we hypothesized that the compensatory NOS isoform is Ca^2+^-dependent, similar to the constitutive isoforms. Indeed, cultured astrocytes from mutant mice exhibited significantly lower NOS activity compared with cultured astrocytes from control mice when calcium was omitted from the medium (27.8±28.03 and 106.6±55.8 nmole nitrite respectively, p = 0.01).

**Figure 5:**
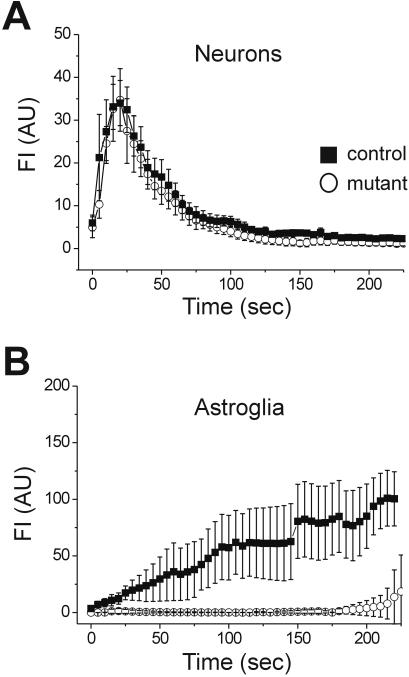
Mutant astroglial NOS activity is dependent on Ca^2+^ release from internal stores. The slices were incubated for 45 minutes in thapsigargin (5 µM) to deplete the Ca^2+^ internal stores. Single cells FI is plotted over time of illumination, and data are expressed as mean±SEM. (A) Putative neurons identified by punctate DAF-2DA fluorescence were not affected by thapsigargin in either mutant (closed squares, 32 cells, 6 slices) or control (open circles, n = 27 cells, 7 slices) mice. (B) Astroglial diffuse fluorescence displayed normal kinetics in control, thapsigargin-treated slices (n = 19 cells, 7 slices), but was almost completely abolished in slices from mutant mice (n = 2 cells, 6 slices).

Since intracellular Ca^2+^ elevation in astrocytes occurs, for the most part, through mobilization from internal stores [21]–[24] we depleted the internal Ca^2+^ stores by incubating the slices for 45 minutes in thapsigargin (5 µM), an inhibitor of the endoplasmic reticulum Ca^2+^-ATPase. The neuronal, punctuate DAF-2DA fluorescence in either mutant or control slices was not affected by the thapsigargin pre-incubation (Fig 5A), in agreement with data showing that neuronal NO production depends mainly on Ca^2+^ entering through NMDA channels [25], [26]. The diffuse astroglial fluorescence in control slices was also unaffected by thapsigargin, and multiple cells could be distinguished in each visual field at time points later than 120 seconds following the start of illumination. In contrast, thapsigargin almost completely blocked the astrocytic fluorescence in slices from mutant mice (Fig 5B), a result which is consistent with a role for intracellular calcium stores in mediating NOS activity in these cells.

### Basal NOS activity in the mutant is increased

**Figure 6:**
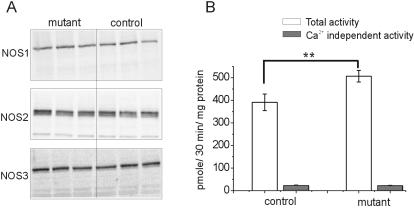
NOS proteins are conserved in the mutant neocortex, but NOS activity is increased. (A) Example of western blot analysis from 3 control and 3 mutant mice neocortex, demonstrating tight conservation of all 3 NOS proteins. (B) The results of NOS radioenzymatic assay reveal an increase in the total NOS activity in mutant mice neocortex (open bars, p = 0.01). The Ca^2+^-independent NOS fraction (gray bars) did not differ between the two mice strains. Data from 3 animals of each strain is displayed as mean±SD.

We next used western blot analysis to examine whether the expression of either of the constitutive NOS isoforms, NOS1 or NOS3, was increased in the mutant neocortex. To test for expression of the mutated protein we used antibodies specific against the NOS2 protein N-terminus. We found tight conservation of the total and relative expression of all three NOS isoforms in the NOS2 mutant, with protein ratios between mutants and control neocortex measuring 88±16% for NOS1, 106±17% for NOS2, and 103±17% for NOS3 (p>0.05, n = 6 animals of each strain, Fig 6A). Immunohistochemistry against NOS1 or NOS3 and glial fibrillary acidic protein, a classic marker for astroglia, did not reveal any co-localization (not shown). Thus, no evidence was found for a compensatory increase in expression of the NOS1 or NOS3 isoforms in astroglia of the mutant mouse. Moreover, the total protein level of NOS isoforms is unchanged, indicating that the increase in basal NOS activity seen by NO fluorescent imaging in the mutants cannot be explained by a change in isoform expression.

The use of DAF-2DA fluorescence for establishing NO concentrations is complicated by its reactivity to peroxynitrite [18] and its dependence on light intensity [27]. Therefore, to compare the basal NOS activity between the neocortex of mutant and control mice, we used an independent method which measures directly the conversion of radiolabeled L-arginine to L-citrulline [28]. Consistent with the imaging data, we found a 30% increase in the total NOS activity in brains of mutant mice, compared with controls (avg. activity 506.8±25.9 and 391.2±36.8 pmole/30 min/mg protein respectively, p = 0.01, Fig 6B). The Ca^2+^-independent NOS fraction was measured by omitting Ca^2+^ from the reaction buffer. This fraction did not differ significantly between the two mice strains, and in both cases it comprised less than 10% of the total NOS activity. Taken together, these data suggest that the NOS2 mutation led to an increase in the basal levels of Ca^2+^-dependent NOS activity.

### Mutant mice exhibit a behavioral phenotype

**Figure 7:**
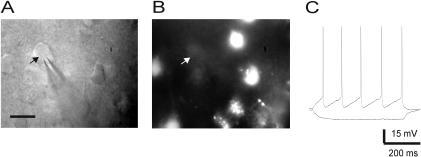
Pyramidal neurons do not exhibit DAF-2DA fluorescence. (A) IR/DIC image of a recorded pyramidal neuron (arrow) and the patch pipette. Scale bar = 25 µm. (B) Fluorescence image of the recording region, revealing fluorescent cells other than the recorded one. The arrow marks the location of the recorded neurons as in (A). (C) Example traces from the recorded neuron in response to injected current pulses. The firing pattern is typical of a regular-spiking pyramidal neuron.

We suspected that the alteration in NOS activity in the mutants' astrocytes may have affected neighboring neurons. Studies using immunohistochemistry reveal that in the neocortex, only a small subpopulation of GABAergic interneurons express the isoform NOS1 [29], [30]. In agreement with these data, pyramidal neurons recorded in DAF-2DA-loaded slices from either mutant or control mice did not exhibit any fluorescence (Fig. 7). Nevertheless, pyramidal neurons from mutant mice exhibited significantly higher input resistance and longer membrane time constant than neurons from control mice, and were similar in all other electrical parameters examined (table 1).

**Table 1:** Comparison of electrophysiological properties between pyramidal neurons of mutant and control neocortex.

	Vm (mV)	Ri (MΩ)	τm (ms)	Spike Amp (mV)	Spike width, 1/2 amp (ms)
Mutant (n = 12)	−71.0±4.3	311.2±91.7^*^	23.3±10.2^**^	72.1±9.1	1.7±0.5
Control (n = 13)	−70.1±4.08	237.7±90.8	15.7±4.9	76.1±8.3	1.4±0.4

Pyramidal neurons were recorded in layer 5 of the primary somatosensory cortex.

Data is expressed as mean±SD. ^*^-p = 0.028; ^**^-p = 0.013, student t-test.

**Figure 8:**
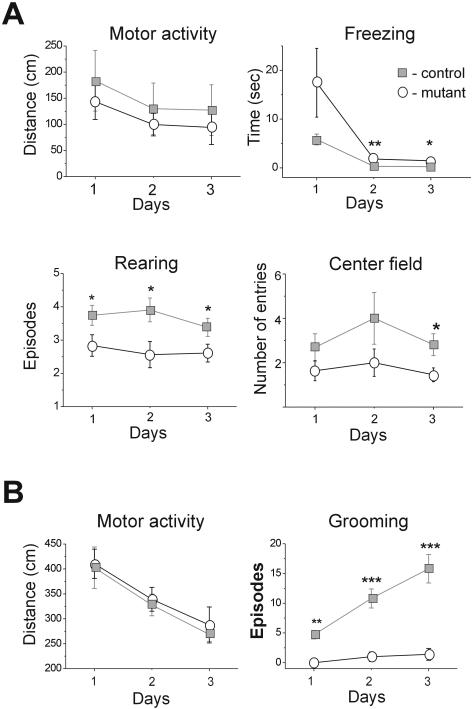
Mutant mice differed from their controls in stress related aspects of exploratory behavior. (A) Control mice (n = 16,) were compared to mutant mice (n = 11, open circles) in the Open-Field test. The general motor activity was measured as the total distance traveled during the test. The Freezing was measured as the latency to escape from the center of the field. Rearing and entries to the center of the field episodes were counted. (B) Example parameters from the results of the Hole-Board test are displayed. The motor activity is measured as the total distance traveled during the test. Grooming episodes are counted (control-n = 6, gray closed squares controls; mutants-n = 9, open circles). For all tests, data is displayed as mean±S.E.M. Significant difference between the groups on a specific day are marked: *-p≤0.05; **-p≤0.01; ***-p≤0.001.

Assuming that these single neuron modifications express a developmental adjustment, we next examined whether more aspects of neuronal activity have been modified leading to detectable behavioral changes. NOS2 mutant mice had normal reflexes and no apparent motor deficit. Testing their basic exploratory behavior, we found no differences between control and mutant mice in their general activity, both in the Open-Field and the Hole-Board tests (Fig 8A,B). In contrast, clear differences between the groups were observed in stress-related parameters. For example, grooming during Open Field and Hole Board tests was almost completely suppressed in mutant compared to control mice (Fig 8B, p = 0.001), while in the safety of their home cages, mutant mice displayed normal grooming behavior. With repeated Hole Board testing grooming increased in both groups, but control mice increased their grooming periods more than mutant mice such that the difference between the groups became even more pronounced (Fig 8B, Open Field, p<0.001; Hole Board, p<0.001). Furthermore, mutant mice displayed increased freezing when initially placed in the center of a square-shaped Open Field (p = 0.002), reared less (p = 0.008), and ventured less into the center of the field (p = 0.039) than control mice (Fig 8A).

**Figure 9:**
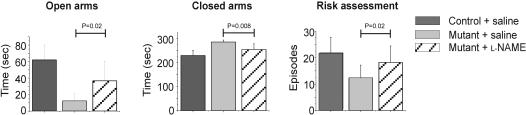
Mutant mice exhibit increased in stress-related parameters on the Elevated Plus maze. Control mice (n = 13, dark gray bars) were compared to mutant mice (n = 10, light gray bars) on the Elevated Plus maze. The time spent in the open or closed arms was measured. Risk assessment designates the combined number of stretch/attend postures and head dipping episodes. Systemic injection with the NOS inhibitor L-NAME (50 mg/kg i.p., n = 10) 10 hours prior to testing improved the performance of mutant mice on the Elevated Plus maze, resulting in significant differences between them and saline-injected animals (n = 11). Control animals were injected with saline only (n = 11), and their motor behavior was not significantly different from non-injected animals.

The mice were also tested in the Elevated Plus maze, a standard test for fear and anxiety. Mutant mice fully avoided the open arms (p = 0.003), remained longer in the closed arms (p = 0.004), and exhibited significantly less head-dipping and stretch/attend postures, behavior which is commonly interpreted as “risk assessment” (p = 0.001). To examine whether increased NOS activity could have contributed to the development of this heightened anxiety, the performance of the mutant mice on the Elevated Plus maze was examined 10 hours following systemic administration of L-NAME (50 µM/kg). At this time point, the effect of L-NAME on arterial blood pressure is expected to be over, while brain NOS activity is still reduced by about 30% [31]. When compared to saline-injected mutant mice, the L-NAME treated mice spent significantly more time in the open arms (p = 0.02), less time in the closed arms (p = 0.008), and increased the frequency of their risk assessment behavior (p = 0.02). Overall, the performance of L-NAME treated mutant mice on the Elevated Plus maze was comparable to that of control mice (Fig 9). Together these data uncover a distinct behavioral phenotype according to which the NOS2 mutant mice are more susceptible to stress, a trait likely to be related to an increased basal NOS activity in their CNS.

## Discussion

Genetically engineered mice with interruptions of the specific NOS genes were developed to help in elucidating the role of NO and the NOS isoforms [10], [11]. However, it has been obvious for some time now that altered genes might affect many developmental processes, and compensatory mechanisms may be activated in knockouts. We demonstrate here intense NO production by astroglia in brains of homozygous NOS2 mutant mice. The data presented is strongly indicative of a constitutive, alternative pathway, which resulted in increased levels of basal NOS activity. In addition, we found that the astrocytic mutation instigated neuronal modifications: 1) neocortical pyramidal neurons exhibit mild but significant changes in their membrane properties, and 2) the mutant mice display a distinct behavioral phenotype.

### The biochemical modifications

While the neuronal DAF-2DA fluorescent response in slices from mutant and control mice was identical on all parameters of analysis, several findings clearly discerned the astrocytic response in mutant from control slices: 1) the fluorescence developed faster and reached higher intensity in mutant slices due to a large number of fast-reacting cells, 2) the fluorescence in mutant astroglia was insensitive to the NOS2 inhibitor 1400W, while this compound completely abolished it in control slices, and 3) astroglial fluorescence in the mutant, but not in the control, depended on release of Ca^2+^ from internal stores. Although there were no changes in the expression levels of any of the three NOS isoforms, NOS activity in neocortex of mutant mice was considerably increased compared with control neocortex.

The exact nature of the biochemical modifications has not been clarified here. The imaging experiments indicate that the compensatory process occurs mainly in astrocytes. The likely possibility is that the mutated protein is now constitutively expressed and uses an alternative pathway. Another possibility for the underlying mechanism is that higher activity of the other constitutive NOS isoforms is compensating for the reduced activity of the mutated protein. However, we failed to find evidence for the expression of the NOS1 or NOS3 isoforms in mutant astroglia to support this possibility.

Whatever is the nature of the biochemical modifications, their existence needs to be taken into account when evaluating brain function in these mutants. It has been well accepted that under pathological conditions, NOS2 expression in astrocytes increases dramatically over a period of hours and days. Hence, several groups used this same NOS2 mutant strain to examine the role of NO in the long-term outcome of brain insults, assuming lack of astrocytic NO production under these conditions. Most notably, these mice were found to be more resistant to MPTP neurotoxicity, a model for Parkinson's disease [12], [13], [32]. On the other hand, the use of these mutants in different models for Alzheimer's disease has yielded disparate views about the role of NO in this neurodegenerative disease [14], [33]. The conclusions of these studies and specifically their mechanistic explanations need to be re-evaluated in light of these results.

### The behavioral phenotype

Both NOS1 and NOS3 knockout mice exhibit distinct behavioral phenotypes, which are unrelated to other systemic alterations such as changes in their blood pressure [34], [35]. Although used by many researchers for over 10 years, no description of behavioral alterations in NOS2 mutants has been published. Our findings delineate a distinct behavioral phenotype in a NOS2 mutant mouse. Interestingly, an earlier study reported changes in the sleep pattern of these mice [36], supporting the finding of CNS modifications. The association between NO and stress-related behavior has been well documented, and pharmacological studies suggest that NO plays an important role in mediating defensive responses [reviewed in 37]. For example, NO signaling has been demonstrated to contribute to fear conditioning in the lateral amygdala [38], CNS injections of NOS inhibitors induce anxiolytic effects in the Elevated Plus maze [39], [40], whereas flight reactions have been seen after administration of NO donors [41], [42]. Thus, the enhanced stress related behaviors of the NOS2 mutant is consistent with these data and is in accord with enhanced NOS activity. The unique finding in our study is the detection of astrocytes as the likely cellular source for the increased NO in mutant mice, and not neurons as typically assumed.

### The cellular source of NO as neuromodulator

The role of NO as a retrograde messenger mediating presynaptic activity-dependent changes has been investigated intensely. While the involvement of the NO-cGMP pathway is relatively established [e.g. 1], [2], [43], [44], its cellular source remains doubtful. Clearly, identifying the cells producing NO to participate in modulating physiological neuronal activity is of great importance for understanding the mechanisms that control its production and its biological role.

In the neocortex, NOS1 is expressed only by a somatostatin-expressing subtype of GABAergic interneurons [29], [30]. It was demonstrated also in spines of hippocampal pyramidal neurons [45], [46], but not in the neocortex. NOS3 is found mostly in the vascular endothelium, and its expression by neurons has been controversial [47], [48]. Our own imaging data are in agreement with this neuronal NOS distribution: while pyramidal neurons in the hippocampus display DAF-2DA fluorescence [personal observation,49], neocortical pyramidal neurons do not. It has been also proposed recently that NO released from endothelial cells participates in modulating nerve cells [50]. The glial option has been largely ignored as the data existing emphasized the expression of astroglial NOS2 only through gene induction following various damage-causing stimuli. Yet, a recent study using spinal-cord slice preparation demonstrated that synaptic potentiation of the presynaptic afferents was mediated by NO released from glial cells, via mGluR1 activation [51]. The NOS2 mutant provides additional evidence, though indirect, for the involvement of astroglial-derived NO in modifying the activity of large neuronal networks as expressed by the intricate stress related behavior.

In recent years it has become increasingly apparent that astrocytes maintain dynamic reciprocal communication with neurons, and may contribute to the regulation of neuronal activity. As part of this concept, the synaptic structure is viewed as the ‘tri-partite synapse’, in which astrocytes play an active role in modulating synaptic transmission [52]–[54]. We speculate that the biochemical compensatory changes in the NOS2 mutant brain described here reflect an essential role for astrocytic NO production, and that astrocytic-produced NO participates in neuronal modulation in the normal brain.

## Materials and Methods

### Animals

Homozygous mice for the *Nos2^tm1Lau^* targeted mutation (NOS2-deficient mice, B6;129P2-*Nos2^tm1Lau^*/J) and their wild-type control (B6;129PF2/J) were obtained from Jackson Laboratories (Bar Harbor, ME). Animals were healthy, and handled routinely under standard conditions of temperature, humidity and a 12 h light/dark cycle, with free access to food and water. All experiments were approved by the Ben-Gurion university committee for the ethical care and use of animals in experiments.

### Imaging and recording in slices

Mice (14–21 days old) were deeply anesthetized with pentobarbital, decapitated, and their brains quickly removed into cold (5°C) physiological solution. Selective astrocytic labeling with SR101 (50 µM in ACSF, Sigma) was done by pressure injection into the neocortex using a picospritzer (General Valve corp.) about 2 minutes prior to decapitation. Neocortical slices (300–400 µm thick) were cut with a vibratome (Campden Instruments, London) and kept in a holding chamber at 36°C for at least 1 hour before any treatment [55], continuously bubbled by 95%O_2_-5%CO_2_. The bathing and superfusing solution contained (in mM): 124 NaCl, 3.5 KCl, 2 MgSO_4_, 1.25 Na_2_HPO_4_, 2 CaCl_2_, 26 NaHCO_3_, 10 dextrose, and was saturated with 95%O_2_-5%CO_2_ (pH 7.4). For NO-imaging experiments, the slices were incubated in Diaminofluorescein–2 Diacetate (DAF-2DA, 2 µM, Calbiochem, La Jolla, CA) for 10 minutes, and then transferred to a slice chamber mounted on an upright fluorescent microscope equipped with IR/DIC optics (Nikon physiostation EC-600), where they were kept at 30–32°C and constantly perfused. Illumination was done with a fluorescent light source (100W mercury lamp), via a Nikon filter (excitation wavelength 450–490 nm, emission wavelength 520 nm), using 60× water immersion objective. Imaging was done using a black and white CCD camera with integrating frame grabber control unit (CCD-300IFG, Dage-MTI, USA), integrating 16 frames for each image. Confocal imaging was done from live slices using a C1si spectral confocal system. The perfusion chamber was mounted on a Nikon FN1 upright microscope equipped with a 1.0 NA water-dipping objective. DAF-2DA images were acquired using the 488 nm line of a 65 mW Argon laser, and SR101 was imaged with a 543 nm HeNe laser.

Fluorescence intensity (FI) was measured off-line using J-image software (Wayne Rasband, NIH). Two types of analysis were carried out. We measured FI of single cells in focus by defining the area of analysis when cells' somata were clearly separable. Otherwise, we measured the FI of the whole visual field. The background was subtracted in both cases.

Whole-cell recordings were performed from neurons and glia with patch pipettes (3–5 MΩ), containing (in mM): 125 K-gluconate, 2 MgCl_2_; 10 HEPES; 10 EGTA; 5 NaCl; 2 Na_2_ATP, pH 7.2, 280 mOsm. Voltages were recorded using patch-clamp amplifier (AxoPatch 2B, Axon Instruments), digitally sampled at 10 kHz and analyzed off-line using LabView-based software. Series resistance was typically <15 MΩ.

The following drugs were added to the incubating solution at various time intervals before imaging, and the same concentration was maintained in the superfusing solution during imaging, unless noted otherwise: N_w_-Nitro-L-arginine (L-NNA); L–N6- (1–iminoethyl) lysine, 2HCl (L-NIL); N-nitro-L-arginine methyl ester (L-NAME); Thapsigargin, all purchased from Sigma-Aldrich, Israel, and N-[[3 (aminomethyl) phenyl]methyl]ethanimidamide dihydrochloride (1400W) was purchased from Tocris (Bristol, UK).

### Western Blot Analysis

The neocortex was dissected immediately following decapitation, homogenized in ice-cold lysis buffer, and centrifuged at 14,000 rpm for 15 min. The supernatant was collected and assayed for total protein concentration using the Bradford method [56]. Samples containing 50 µg proteins were loaded and the protein size was separated in 7.5% SDS polyacrylamide gel electrophoresis (150 V) along with a set of molecular-weight markers (Bio-Rad, UK). Blots were electrotransferred onto nitrocellulose membrane, blocked with blocking buffer, and then incubated overnight with specific primary polyclonal antibodies against NOS1 (1∶200–1∶500), NOS2 (1∶200–1∶1000) and NOS3 (1∶200), all of them purchased from Santa Cruz Biotechnology, CA. Positive controls from macrophages were used for NOS2. Blots were exposed to horseradish peroxidase-conjugated anti-rabbit IgG secondary antibody (1∶7,500, Santa Cruz Biotechnology), and immunoreactivity was visualized using ChemiImager technology, following manufacturer's instructions (Alpha Innotech Corporation, San Leandro, CA).

### NOS activity assays

NOS catalytic activity was assayed by measuring both the Ca^2+^-dependent and the Ca^2+^-independent conversion of [^3^H]arginine to [^3^H]citrulline as described by Przedborski et al. [28], using NOS activity assay kit (Cayman chemicals, Ann Arbor, MI). In brief, brains of 21 days old mice were quickly removed and the neocortex was homogenized in 1 ml buffer containing 25 mM Tris-HCL (pH 7.4), 1mM EDTA and 1mM EGTA. Samples were incubated at room temperature for 30 min in the presence of [^3^H]-arginine (1 µCi/µl; Amersham, UK) and cofactors. The reaction was terminated by the addition of stop buffer containing 50 mM HEPES (pH 5.5) and 5 mM EDTA. To determine the relative fraction of calcium-independent NOS activity, calcium was omitted from the reaction mixture in some samples and EGTA (1 mM) was added. [^3^H]-citrulline was quantified by liquid scintillation counting of the eluate, and the counts per minute for all samples were averaged and corrected with respect to the background radioactivity. NOS activity was expressed as pmol [^3^H] L-citrulline/30 min/mg protein.

To compare NOS activity between cultured astrocytes from mutant and control mice, nitrite concentrations were determined using an assay kit based on the reaction of nitrite with 2,3-diaminonaphthalene to form the fluorescent product, 1-(*H*)-naphthotriazole (*Calbiochem*, Cat. No. 482655), following standard instructions. The reading was performed with wavelength of 365 nm excitation, and 450 nm emission.

### Behavioral testing

Control and mutant adult male mice were transferred to the lab prior to testing, and remained there throughout the testing period. The animals were neither handled nor habituated to the behavioral apparatus before the initial test session. Tests were carried out during the light period (12:00–16:00). Mouse behavior was recorded from a centrally placed video camera located 0.5 m away from the apparatus under dim light. A hole-board was used to examine explorative behavior. Each animal was placed in the center of the board and its behavior videotaped for 5 minutes, and the test was repeated for three successive days. Head dipping and grooming episodes, as well as distant travel on the board were recorded off the videotapes. For the Open-Field test, each mouse was placed in the center of the field and its latency to escape to any corner was recorded. The behavior was videotaped for 10 minutes, and the following parameters were recorded: the field was divided into 25 squares (20 cm×20 cm each) and the total (center and peripheral) squares crossed, the distance traveled, the number of entries into the center of the field, and the number of rearing and grooming episodes. The test was repeated over a total of three consecutive days. An Elevated Plus maze with two open and two closed arms (each 30 cm long×5 cm wide) was used for a single 10-minutes exposure. The time spent in the open arms and duration of grooming were recorded off the videotapes as well as the number of partial (2 legs) and full (4 legs) entries into the open arms, the number of stretch/attend postures, and the head dipping over the side of the open arms (the combined last two parameters were interpreted as “risk assessment” behavior). Two-way analysis of variance (ANOVA) with repeated measure was used to examine group differences over days (when tested), with a significance level of 0.05.
